# Enhanced plant bottom-up histone proteomics

**DOI:** 10.1093/jxb/erag100

**Published:** 2026-02-24

**Authors:** Palina Ryzhaya, Pavlína Pírek, Radomír Pech, Miroslava Karafiátová, Jan Bartoš, Zbyněk Zdráhal, Gabriela Lochmanová

**Affiliations:** Mendel Center for Plant Genomics and Proteomics, Central European Institute of Technology, Masaryk University, Brno 625 00, Czech Republic; National Centre for Biomolecular Research, Faculty of Science, Masaryk University, Brno 625 00, Czech Republic; Mendel Center for Plant Genomics and Proteomics, Central European Institute of Technology, Masaryk University, Brno 625 00, Czech Republic; Mendel Center for Plant Genomics and Proteomics, Central European Institute of Technology, Masaryk University, Brno 625 00, Czech Republic; Institute of Experimental Botany of the Czech Academy of Sciences, Centre of Plant Structural and Functional Genomics, Olomouc 779 00, Czech Republic; Institute of Experimental Botany of the Czech Academy of Sciences, Centre of Plant Structural and Functional Genomics, Olomouc 779 00, Czech Republic; Mendel Center for Plant Genomics and Proteomics, Central European Institute of Technology, Masaryk University, Brno 625 00, Czech Republic; National Centre for Biomolecular Research, Faculty of Science, Masaryk University, Brno 625 00, Czech Republic; Mendel Center for Plant Genomics and Proteomics, Central European Institute of Technology, Masaryk University, Brno 625 00, Czech Republic; National Centre for Biomolecular Research, Faculty of Science, Masaryk University, Brno 625 00, Czech Republic; Université Clermont Auvergne, France

**Keywords:** Crop plants, flow cytometry, histone derivatization, maize, mass spectrometry, post-translational modification, proteomics, trimethylacetic anhydride, *Zea mays*

## Abstract

The correct tools for characterization of histone proteoforms are essential for deciphering plant epigenetic mechanisms and their subsequent application in biotechnology. Insights into the epigenetic landscape of plant chromatin can be advanced using bottom-up proteomics. MS analysis of histone peptides relies on careful sample preparation, including chemical derivatization of amine groups prior to MS to improve their chromatographic behaviour during nanoHPLC separation. Characterizing histones in plant tissues remains especially challenging due to the presence of diverse, species-specific compounds that interfere with MS analysis. In this study, we evaluated the impact of different protocols for the preparation of histones from maize (*Zea mays*) leaves on the quality of MS data. We were able to enhance the MS-based plant histone analysis protocol by combining chemical derivatization using trimethylacetic anhydride with enzymatic digestion with a novel protease, Arg-C Ultra. In addition, fluorescence-assisted cell sorting proved to be effective in isolating pure histone samples without the need for protein purification by precipitation. Our proposed new workflows produce highly pure histone extracts that are suitable for quantitative analysis of post-translational modifications and variant composition. They therefore offer a powerful tool for investigating epigenetic patterns and their dynamics in agriculturally important crops.

## Teaser

Refined plant histone preparation protocols based on chemical labelling improve LC-MS/MS analysis and enable expanded epigenetic profiling of post-translational modifications in maize.

## Introduction

Epigenetic mechanisms play key roles in the regulation of plant growth, development, and adaptation to adverse environmental conditions (Tresas *et al*., 2025). A growing number of studies have shown that modulation of these regulatory processes can contribute to sustainable food production by increasing yield and nutrient content of crops, making ‘epi-breeding’ a valuable alternative to traditional genetic manipulations (reviewed by Fang *et al*., 2023). Gene expression is closely associated with the specific patterns of histone modifications within a given genomic region. Some histone marks serve as transcription activators (such as H3K4me3, H3K36me3, and H3/H4 acetylation) while others are strongly associated with gene silencing (such as H3K27me3 and H3K9me3) (Kouzarides, 2007; Ramirez-Prado *et al*., 2018). For instance, the genes responsible for the morphological traits of maize (*Zea mays*) during development are controlled by the histone deacetylase HDA108, a ubiquitously expressed member of the Rpd3/HDA1 histone deacetylase family, that modulates gene expression by altering the acetylation levels of histones H3 and H4 (Forestan *et al*., 2018). A significant increase in H3K9 and H4K5 acetylation levels accompanied by a decrease in H3K9me2 has been reported in the context of heat stress (Yu *et al*., 2020).

From this perspective, mapping the epigenetic profile of economically important crops is crucial. Although research into epigenetic modifications in plant species will never rival that in animal studies, plant epigenetics is now attracting considerable interest, with our knowledge increasing rapidly. Pilot species have included maize, a versatile, multipurpose crop that is currently the world’s leading cereal crop in terms of total yield (FAO, 2024), and is consequently an intensively studied plant model. It is primarily used as an animal feed, but is also important as a food crop and hence for ensuring food security worldwide. Despite the growing interest in the epigenetic regulation of maize (reviewed by Yu *et al*., 2020), we are still far from deciphering its intricate mechanisms and hence there is a need for innovative approaches.

Continuing methodological advances in bottom-up proteomics offer new insights into histone epigenetics. MS analysis delivers a comprehensive quantitative perspective on histone post-translational modifications (PTMs) and sequence variants at the system-level (Thomas *et al*., 2020). The usual pipeline for mammalian histone preparation prior to MS involves acidic extraction of histones followed by protein precipitation to ensure removal of contaminants and a high yield of pure histones. Due to the frequent occurrence of lysine and arginine residues in the amino acid sequence of histones, chemical derivatization of amine groups is performed before trypsin digestion to prevent the formation of short hydrophilic peptides and their subsequent loss. Another round of derivatization, usually performed on the peptide level to label newly formed amine groups, enhances chromatographic separation (Garcia *et al*., 2007). The retention and resolution of labelled peptides on analytical chromatography columns vary depending on the derivatization reagent used. Propionic anhydride (Prop) is the most commonly used agent for mammalian histone derivatization, often complemented by labelling with phenylisocyanate at the peptide level (Maile *et al*., 2015). The enhanced separation of frequently occurring isobaric modified histone peptides and their quantification from the MS1 level can be achieved using trimethylacetic anhydride (TMA) (Kuchaříková *et al*., 2021). Recently, protein digestion with Arg-C Ultra, a novel proteinase with increased arginine-cleavage specificity, followed by TMA derivatization at the peptide level has been shown to facilitate the preparation of mammalian histones (Ryzhaya *et al*., 2025). Current LC-MS/MS techniques for mammalian histone preparation and measurement are in need of further refinement and development, but nonetheless they have been widely adopted by the scientific community. These methods are now routinely used to dissect changes in histone PTMs in the context of diseases or medical treatments (Lu *et al*., 2021; Noberini *et al*., 2022; Yao *et al*., 2024).

However, analysing plant histones using MS is less straightforward. The complex composition of plant tissues—particularly the presence of diverse secondary metabolites, polysaccharides, polyphenols, pigments, lipids, organic acids, and abundant non-histone proteins—can interfere with histone preparation, affecting both the purity and yield of the peptide mixture. As a result, histone characterization is less commonly performed in plant studies than in mammalian research (Scheid *et al*., 2022), with most plant-based analyses conducted in Arabidopsis, an excellent model organism for studying epigenetic regulation. Zhang *et al*. (2007) laid a valuable foundation for the comprehensive identification of PTMs in core histones and their roles in plant growth and development. MS has also been applied to characterize H2B in Arabidopsis, either for detailed analysis of histone sequence variants (Bergmüller *et al*., 2007) or for comparing PTM profiles between different lines (Nassrallah *et al*., 2018). To enhance detection and improve quantification of post-translationally modified forms by LC-MS/MS, chemical derivatization has been incorporated into plant sample preparation protocols (Johnson *et al*., 2004). Current step-by-step protocols for preparing histones from Arabidopsis consistently include propionylation of amine groups, but differ in their methods for nuclei preparation and sample purification (Kuchaříková *et al*., 2022; Scheid *et al*., 2022; Geshkovski *et al*., 2025).

Previous studies have demonstrated that histones from Arabidopsis are hard to re-dissolve in water after precipitation (Ledvinová *et al*., 2018; Scheid *et al*., 2022), and additional methanol:chloroform clean-up of histone proteins originating from Arabidopsis leaves is required prior to LC-MS/MS (Scheid *et al*., 2022). A filter-aided sample preparation (FASP) procedure coupled with propionylation (hereafter referred to as a FASP-based procedure) established by Ledvinová *et al*. (2018) significantly improved proteomic analysis from as little as 0.5 g of plant tissue, allowing quantification of peptidoforms and determination of the ratio between histone sequence variants in various biological contexts. For instance, comprehensive proteomic characterization of histone PTMs revealed the relationship between phenotypic features and the pattern of histone modifications in Arabidopsis plants with abolished histone deacetylase activities (Lochmanová *et al*., 2019), and a distinct H3.1:H3.3 ratio has been detected in Arabidopsis mutants lacking certain histone chaperones (Kolářová *et al*., 2021). More recently, using the same approach, the interplay between different epigenetic layers has been demonstrated, namely changes in histone PTMs and replacement of histone sequence variants (Pírek *et al*., 2023; Franek *et al*., 2024). Specifically, it was shown that defects in histone chaperone function (e.g. mutations in CHROMATIN ASSEMBLY FACTOR-1 or NUCLEOSOME ASSEMBLY PROTEIN 1) translate into an altered epigenetic landscape, which aids the plant in mitigating internal instability (Franek *et al*., 2024). The FASP-based procedure used in the study even enabled the discrimination and quantification of the subtypes of histone sequence variants H2A.Z (H2A.Z.8, H2A.Z.9, and H2A.Z.11), H2A.X (H2A.X.3 and H2A.X.5), and H2A.W (H2A.W.6 and H2A.W.7). A study by Pírek *et al*. (2023) revealed that histone variant replacement plays a substantial role in chromatin remodeling during callus induction and propagation, while changes in histone acetylation and methylation levels serve as fine-tuning mechanisms that are important for the activation or repression of specific genes. While the FASP-based plant histone preparation protocol is effective, it yields limited numbers of identified post-translationally modified peptidoforms due to the low hydrophobicity of the propionyl adduct and insufficient resolution of isobaric peptides.

In pursuit of more precise histone profiling in plants, in this study we present MS-based methods that enhance the quantitative analysis of histone peptidoforms and facilitate new discoveries in plant epigenetics. To try to better align the quality of plant histone characterization—specifically the range of quantified peptidoforms—with that achieved in mammalian systems, we incorporated chemical derivatization using TMA into the histone preparation workflow for *Zea mays* leaves. Nuclei purified via flow cytometry were tested as an alternative input to minimize interference from natural plant compounds. Of the seven protocol variants that we tested, we highlight two efficient methods for preparing maize histones for LC-MS/MS analysis. In the first protocol, the extracted histones are precipitated, followed by enzymatic cleavage using Arg-C Ultra, and the peptides are chemically derivatized using TMA. The second, a protocol based on fluorescence-assisted cell sorting, yields pure histone extracts, enabling direct in-solution protein cleavage followed by TMA labelling, and it is advantageous for plants in which the chemical composition of the tissue interferes with any step in the histone sample preparation procedure or subsequent LC-MS/MS measurements. Both protocols provide comprehensive quantitative data on histone marks, offering a basis for analysis of epigenetic regulation in maize.

## Materials and methods

**Fig. 1.**
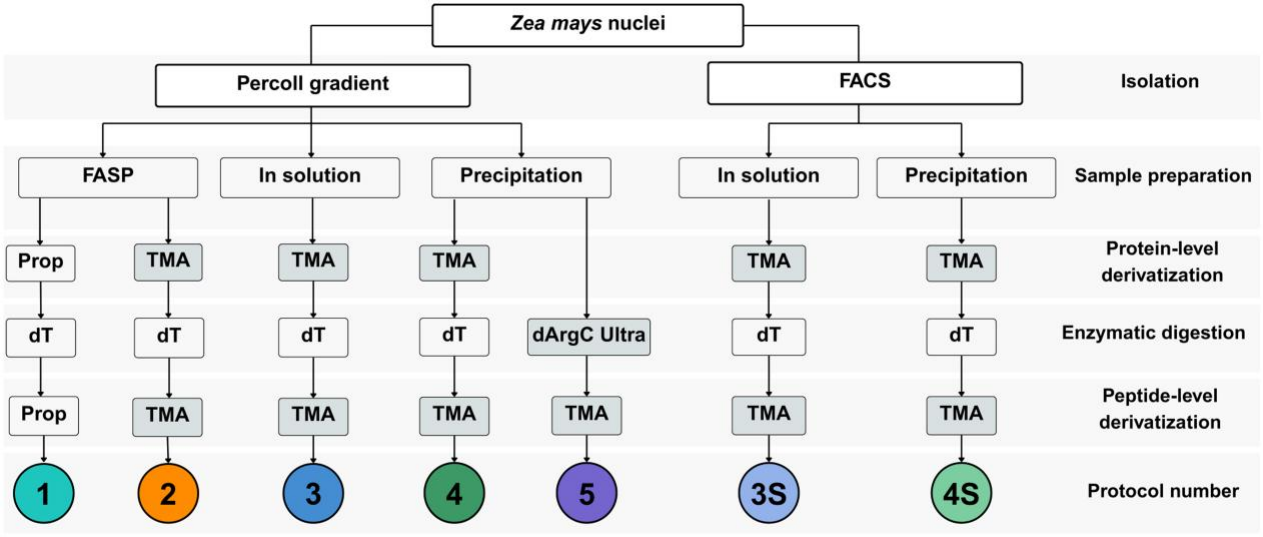
Overview of the seven protocols tested for plant histone preparation in *Zea mays*. Histones were isolated by acidic extraction from either crude (1–5) or sorted nuclei (3S and 4S), and the subsequent steps in the individual protocols are indicated. dT, tryptic digestion; dArg-C Ultra; digestion with Arg-C Ultra; FACS, fluorescence-assisted cell sorting; FASP, filter-aided sample preparation; Prop, propionic anhydride; TMA, trimethylacetic anhydride.

Altogether, seven protocols were tested for plant histone preparation (Fig. 1). Below, common procedures shared across multiple protocols are described, and protocol-specific steps are detailed within each relevant section. Protocols 5 and 3S, which were evaluated as being the preferred methods for histone preparation from maize and other crops, have been made freely available online in a step-by-step format (see Data availability section, below).

### Reagents for histone preparation

Phenylmethylsulfonyl fluoride (PMSF) and Micro BCA™ Protein Assay Kits were purchased from Thermo Fisher Scientific. EDTA was purchased from Bio-Rad. Triton X-100 was purchased from Carl ROTH. Sulfuric and hydrochloric acids were purchased from Penta (Czech Republic). Acetonitrile (ACN) and formic acid (FA) were purchased from Honeywell (NC, USA). Trimethylacetic anhydride (TMA), propionic anhydride (Prop), trichloroacetic acid (TCA), ammonium bicarbonate (AB), SOLu-trypsin dimethylated, and DTT were purchased from Merck. AttractSPE^®^ Tips C18 were purchased from Affinisep (France). Arg-C Ultra was purchased from Promega. Percoll, Protease Inhibitor Cocktail P9599, and Phosphatase Inhibitor Cocktail P5726 were purchased from Sigma-Aldrich.

### Plant material

The seeds of *Zea mays* inbred line B73 used in this study were harvested at the Institute of Experimental Botany in the Czech Republic. Leaves for nuclei isolation were collected from 16-day-old plantlets that had been grown in soil under controlled conditions in growth chambers under 16/8 h light/dark (250 μmol m^–2^ s^–1^) at 24/20 °C.

### Isolation of crude nuclei (Protocols 1, 2, 3, 4, and 5)

Crude nuclei were isolated according to the previously published protocol of Ledvinová *et al*. (2018). The leaves of the plantlets were ground in liquid nitrogen and homogenized in extraction buffer containing 10 mM NaCl, 10 mM 2-(N-morpholino)ethanesulfonate (pH 6.0), 5 mM EDTA, 0.25 M sucrose, 0.6% Triton X-100, 0.2 M spermidine, 100 mM PMSF, 45 mM sodium butyrate, and 20 mM β-mercaptoethanol. The homogenate was filtered through nylon mesh (pore size 162 μm) and centrifuged at 3000 *g* at 4 °C for 10 min. The resulting pellet was washed twice with extraction buffer, resuspended in Percoll buffer (2.4 g of 5× concentrated extraction buffer and 18 g of Percoll), and centrifuged at 4000 *g* at 4 °C for 15 min. Nuclei floating at the surface of the Percoll buffer were collected, washed three times in wash buffer (75 mM NaCl, 10 mM EDTA, 50 mM Tris-HCl pH 8.0), and centrifuged at 3000 *g* at 4 °C for 10 min. The pellet was then incubated in histone lysis buffer (80 mM NaCl, 20 mM EDTA, 1% Triton X-100) supplemented with protease, deacetylase, and phosphatase inhibitors (0.1 mM PMSF, 45 mM sodium butyrate, 10 μl ml^–1^ protease inhibitor cocktail P9599, and 10 μl ml^–1^ phosphatase inhibitor cocktail P5726) for 1 h on ice, centrifuged at 10 000 *g* at 4 °C for 5 min, washed with 50 mM Tris-HCl 200 μl, and centrifuged at 10 000 g at 4 °C for 5 min. Histones were extracted from the chromatin pellet into 250 μl of ice-cold 0.2 M H_2_SO_4_ overnight at 4 °C.

### Isolation of nuclei assisted by fluorescence-assisted cell sorting (Protocols 3S and 4S)

For fluorescence-assisted cell sorting (FACS), maize nuclei were purified using a FACSAria SORP flow cytometer (BD Biosciences) as described previously (Šimková *et al*., 2003). Briefly, the leaves were fixed for 20 min at 4 °C in 2% formaldehyde made in Tris buffer (10 mM Tris-HCl, 10 mM EDTA, 100 mM NaCl, pH 7.5) and washed three times in Tris buffer. The nuclei were released by chopping the leaves with a razor blade in ice-cold lysis buffer [15 mM Tris, 2 mM Na_2_EDTA, 0.5 mM spermine.4HCl, 80 mM KCl, 20 mM NaCl, 0.1% (v/v) Triton X-100, 15 mM mercaptoethanol, pH 9.0]. The crude nuclei suspension was filtered through a 50 µm nylon mesh, stained with DAPI (2 µg ml^–1^), and analysed. In total, 27 batches each of 1 000 000 G1 nuclei were collected on ice into 2.0 ml microtubes containing 10 µl of ddH_2_O. The isolated nuclei were immediately centrifuged at 3000 *g* at 4 °C for 5 min to remove the sheath fluid. Subsequently, 50 μl of 0.2 M H_2_SO_4_ was added to the pellet for lysis and histone extraction. The samples were incubated overnight on a rotator at 4 °C. After centrifugation at 16 100 *g* at 4 °C for 8 min, the supernatant containing the histones was transferred to a new vial, and the samples were stored at −80 °C.

### Protein concentration measurement

Protein concentration was measured using a Micro BCA™ Protein Assay Kit in a microplate. A sample of 1 μl of histone extract in sulfuric acid was diluted 50 times with water, and 50 μl of WR reagent was added. After 2 h incubation at 37 °C, the absorbance was measured at 562 nm in a Biotek Synergy H1 microplate reader (Agilent Technologies).

### Histone precipitation (Protocols 4, 4S, and 5)

A sample of 250 μl of histone extract was precipitated with 250 μl of 50% ice-cold TCA by incubation with shaking at 0 °C for 30 min. The precipitate was collected by centrifugation at 5000 *g* and 4 °C for 30 min, washed once with 50 mM HCl in acetone and twice with pure acetone, and then dried at room temperature.

### Protocol 1: filter-aided histone preparation from crude nuclei coupled with trypsin digestion and propionylation

A sample of 16 µg of histone extract in sulfuric acid was subjected to chemical derivatization. First, pH was adjusted to 8 with NH_4_OH. The derivatization reagent was freshly prepared for each reaction by mixing Prop with ACN in a 1:3 (v/v) ratio. Samples were incubated in a thermomixer at 37 °C for 20 min at 750 rpm, and then concentrated in a vacuum concentrator to 5 μl at 35 °C for 50 min. The samples were then diluted with 50% ACN to a final volume of 20 μl, the pH was adjusted to 8, and the second round of chemical derivatization was performed. Following clean-up and enzymatic digestion using the filter-aided sample preparation described below, the newly released peptide N-termini were again subjected to the chemical derivatization described above, with the addition of 5 μl of propionylation reagent. The samples were then concentrated to dryness in a vacuum concentrator at 35 °C overnight.

### Protocol 2: filter-aided histone preparation from crude nuclei coupled with trypsin digestion and TMA-labelling

A sample of 16 µg of histone extract in sulfuric acid was subjected to chemical derivatization. First, pH was adjusted to 8 with NH_4_OH. For the protein-level TMA derivatization described below, 3 μl of reagent was used. Following clean-up and enzymatic digestion using the filter-aided sample preparation, newly released peptide N-termini were subjected to peptide-level TMA derivatization as described below.

### Protocols 3 and 3S: histone preparation coupled with in-solution trypsin digestion and TMA-labelling

The pH of histones extracted from crude (Protocol 3) or nuclei sorted by FACS (Protocol 3S) was adjusted to 8 with NH_4_OH, and the volume was reduced in a vacuum concentrator to 20 μl at 35 °C for 1 h. For the protein-level derivatization described below, 3 μl of TMA reagent was used. The resulting labelled protein sample was concentrated to 5 μl in a vacuum concentrator at 35 °C for 20 min, and 0.25 µg of SOLu-trypsin in 40 μl of 100 mM AB was added. After 4 h of incubation at 37 °C, another aliquot of 0.25 µg of SOLu-trypsin was added, and the sample was then incubated for 12 h at 37 °C. Newly released peptide N-termini were subjected to the peptide-level TMA derivatization as described below.

### Protocols 4 and 4S: histone preparation including protein precipitation, digestion with trypsin, and TMA-labelling

Precipitated histones from crude (Protocol 4) or sorted nuclei (Protocol 4S) were re-dissolved in 20 μl of water. For the protein-level derivatization described below, 3 μl of TMA reagent was used. The resulting labelled protein sample was concentrated to 5 μl in a vacuum concentrator at 35 °C for 20 min, and 0.25 µg of SOLu-trypsin in 40 μl of 100 mM AB was added. After 4 h of incubation at 37 °C, another aliquot of 0.25 µg of SOLu-trypsin was added, and the sample was incubated for further 12 h at 37 °C. Newly released peptide N-termini were subjected to peptide-level TMA derivatization as described below.

### Protocol 5: histone preparation from crude nuclei including protein precipitation, digestion with Arg-C Ultra, and TMA-labelling

Precipitated histones were re-dissolved in 27 μl of water, and 0.5 M AB (pH 7.5) and 200 mM DTT were added to final concentrations of 50 mM and 10 mM, respectively. Then 0.1 µg of Arg-C Ultra was added, the mixture was incubated at 37 °C for 90 min, the same amount of Arg-C Ultra was added again, followed by another 90 min incubation. Then 10 μl of 100% (v/v) ACN was added to the sample and the pH was adjusted to 8 with NH_4_OH. For the peptide-level derivatization described below, 2.25 μl of TMA reagent was added to each sample.

### Filter-aided histone preparation (Protocols 1 and 2)

Protein-level derivatized samples were diluted with 300 μl of 8 M urea in 0.1 M Tris-HCl (pH 8.5), placed in YM-10 Microcon filter unit (Merck), centrifuged at 14 000 *g* at 20 °C for 30 min, and washed twice with 200 μl of 8 M urea and three times with 100 μl of 100 mM AB. Then, 50 μl of 100 mM AB was added with 400 ng of SOLu-trypsin dimethylated and incubated at 37 °C overnight. The samples were centrifuged three times at 14 000 *g* at 20 °C for 10 min, the second and third time with the addition of 50 μl of 100 mM AB. The samples were concentrated to 20 μl in a vacuum concentrator at 37 °C.

### Chemical derivatization of histones using trimethylacetic anhydride

The TMA derivatization reagent was prepared by mixing trimethylacetic anhydride with ACN in a 1:3 (v/v) ratio. The reagent was prepared fresh for each reaction.

For protein-level derivatization (applied in Protocols 2, 3, 3S, 4, and 4S), the samples were incubated with the reagent for 5 h at room temperature while shaking, followed by a repeated derivatization step including 16 h incubation, and then subjected to two rounds of microwave-assisted derivatization. The sample volume was reduced in a vacuum concentrator at 35 °C to ∼5 μl, then diluted with 50% (v/v) ACN to a final volume of 12 μl. The sample was incubated in a microwave oven for the first round of microwave-assisted derivatization. This consisted of three sub-cycles with the following steps: adjustment of the sample pH to 8 with NH_4_OH, addition of 3 μl of TMA reagent, and two incubations of 1 min each in the microwave oven at 350 W with a short centrifugation (30 s at 2000 *g*) between them. The sample was then concentrated to 3–5 μl in a vacuum concentrator at 35 °C, 20 μl of 50% ACN was added, and the second round of microwave-assisted derivatization was carried out with the same protocol. The resulting TMA-derivatized protein sample was concentrated to 5 μl in a vacuum concentrator at 35 °C.

Two rounds of peptide-level derivatization were performed following digestion of either derivatized proteins with trypsin (Protocols 2, 3, 3S, 4, and 4S) or non-derivatized proteins with Arg-C Ultra (Protocol 5), using the microwave-assisted derivatization protocol described above with the addition of 5 μl of TMA reagent. Then, the samples were dried in a vacuum concentrator at 35 °C overnight.

### Sample desalting (all protocols)

The dried samples were reconstituted in 50 μl of 0.1% trifluoroacetic acid (TFA). Desalting was performed using AttractSPE^®^ Tips C18. Peptides were sequentially eluted with 2× 10 μl of 0.1% TFA in 50% ACN and 2× 20 μl of 0.1% TFA in 75% ACN. The eluates were transferred to a LC vial, concentrated to 10 μl, and acidified with FA to a final concentration of 1%.

### LC-MS/MS analysis

Samples were analysed in a random order using an UltiMate 3000 RSLCnano liquid chromatograph coupled to an Orbitrap Fusion Lumos Tribrid mass spectrometer (Thermo Fisher Scientific). A quantity of 200 ng of each peptide mixture was injected, concentrated on an online trap column (cartridge type μPrecolumn, 300 μm ID, 5 mm long; Thermo Fisher Scientific) packed with C18 PepMap100 (5 μm particles, 100 A), and separated on an Aurora C18 analytical column (25 cm long, 75μm inner diameter, 1.7 μm particles; Ion Opticks, Australia). The trap and analytical columns were set at 25 °C and at 50 °C, respectively. The mobile phases used for the gradient elution consisted of a binary mixture of 0.1% FA in water (A) and 0.1% FA in 80% ACN (B). Peptides were eluted with a 120 min gradient at a 300 nl min^−1^ flow rate and the content of B rising from 3% (0–4 min), 3–42% (4–107 min), 42–80% (107–115 min), and followed by an isocratic wash of 80% B (115–120 min). Before sample injection into the loop, the trapping and analytical columns were equilibrated with 99:1 (mobile phase A:B) at a flow rate of 400 nl min^–1^. The analytical column outlet was directly connected to a Digital PicoView 550 ion source equipped with an Active Background Ion Reduction Device (ESI Source Solutions, MA, USA).

Mass spectra were acquired in data-dependent acquisition (DDA) mode using 350–2000 *m*/*z* survey scans at a resolution of 120 000 (at *m*/*z* 200) with an automatic gain control target setting of 4×10^5^ and a maximum injection time of 500 ms. Precursors with charge states from 2+ to 7+ and intensity above 1×10^4^ were subjected to higher energy collisional dissociation fragmentation with a normalized collision energy of 30%. Once fragmented, precursors were excluded for 30 s before the next fragmentation. Precursors were isolated by quadrupole with a 1.2 *m*/*z* isolation window. Tandem mass spectra were obtained using 30 000 resolution (at *m*/*z* 200). Ions were accumulated for a target value of 5×10^4^ or 500 ms injection time. The cycle time between master scans was 2.5 s.

### Database search, data evaluation, and statistical analysis

The MS raw data were searched against the modified cRAP contamination database (based on http://www.thegpm.org/crap/; 112 sequences), an in-house histone *Zea mays* database (229 protein sequences generated from UniProt), and the UniProt KB *Zea mays* database (taxon ID 4577, https://www.uniprot.org/taxonomy/4577; 39 228 sequences). The searches were conducted using an in-house Mascot search engine (v2.6.2; Matrix Science, MA, USA) via the Proteome Discoverer software (v2.2.0.388; Thermo Fisher Scientific). The mass error tolerance for precursor ions was set at 10 ppm for cRAP and 7 ppm for the *Zea mays* database searches. For MS/MS fragment ions, the tolerances were 0.05 Da for cRAP and 0.03 Da for the databases. Enzyme specificity was configured to semi-Arg-C, allowing for two missed cleavages across all databases. Variable modifications for each database were as follows. cRAP: deamidation (N and Q), oxidation (M), and propionylation or trimethylacetylation (peptide N-terminal region, K, S, T, and Y). Histone *Zea mays* in-house database: acetylation (protein N-terminal region and K), phosphorylation (S, T), methylation (K, R), dimethylation (K), trimethylation (K), and trimethylacetylation or propionylation (peptide N-terminal region, K, S, T, and Y). UniProt KB *Zea mays* database: acetylation (protein N-terminal region), trimethylacetylation or propionylation (peptide N-terminal region, K, S, T, and Y). Peptides identified using the in-house database were refined by a fixed-value peptide-spectrum match validator (delta Cn<0.05), followed by filtering highly confident peptides using Mascot parameters set to Rank 1, expectation value <0.01, and ion score ≥30. Peptides identified using the UniProt database were refined by Percolator (Cn<0.05, false discovery rate <0.01). Selected identified peptides were manually inspected, and their quantities were determined and manually validated using Skyline-daily software (24.1.1.202; https://skyline.ms/home/software/Skyline/) based on peak areas in extracted ion chromatograms (EICs), including identification alignment across the raw files based on retention time and *m*/*z*.

The quantitative evaluation of histone peptides was performed in the KNIME Analytics Platform (https://www.knime.com/knime-analytics-platform) with R scripts. The precursor peak areas of histone peptidoforms were log_2_-transformed and their relative abundances were calculated as the ratio of individual EIC precursor peak area to the sum of the EIC areas of all forms of the respective peptide sequence. Peak areas for all identified forms were treated as compositions and combined using geometric means.

## Results

The implementation and adaptation of existing MS-based methodologies is necessary if we are to advance our understanding of plant histone epigenetics. To enhance the detection and quantification of a wide range of histone peptidoforms, we introduced chemical derivatization with TMA into plant histone preparation protocols. The TMA-labelling performance was examined using histone extracts prepared from maize nuclei isolated from leaves using either a Percoll gradient (crude nuclei) or FACS (sorted nuclei). In total, we compared the results of seven histone preparation protocols (Fig. 1). Derivatization at the protein level prior to tryptic digestion was applied in the filter-aided histone preparation from crude nuclei coupled with Prop- or TMA-labelling (Protocols 1 and 2), the extraction of histones from crude or sorted nuclei followed by in-solution TMA-labelling (Protocols 3 and 3S), and in the precipitation of histones from crude or sorted nuclei followed by in-solution TMA-labelling (Protocols 4 and 4S). In contrast, a protocol that omitted protein-level derivatization involved precipitation of histones from crude nuclei, followed by Arg-C Ultra digestion and TMA-labelling (Protocol 5). Protocol 1 served as an example of a standard procedure currently used for plant histone preparation (Ledvinová *et al*., 2018).

### Quality control of isolated nuclei and histone extracts

For all protocols, the input material was derived from nuclei isolated from a single 16-day-old plant. Sorted nuclei displayed markedly higher purity compared with crude nuclei, which showed contamination by plant tissue debris (Supplementary Fig. S1A). The median total protein amounts from biological replicates of histone extracts were 10 μg (crude) and 14 μg (sorted) in neutralized histone samples, and 2 μg (crude) and 6 μg (sorted) in precipitated histone samples.

**Fig. 2.**
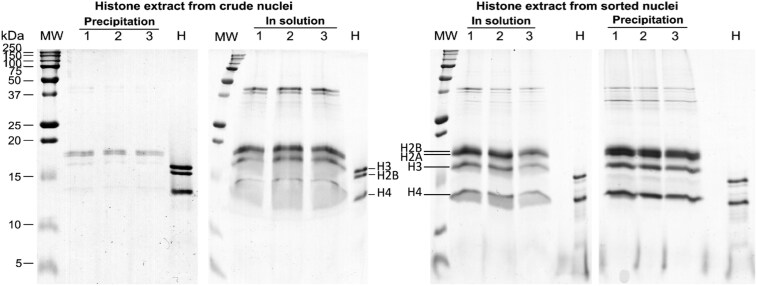
Quality control of histones extracted from crude or sorted nuclei. Histone proteins are found in the region of 10–20 kDa. Each band represents a mixture of histone sequence variants and the predominant component of each band as identified by LC-MS/MS is indicated: H4 (UniProt accession nos P62787/Q41811), 11.4–11.6 kDa; H3 (P69246/B6TTP0/B6SIV8), 15.3 kDa; H2A (B4FU52/B6SJU3/P40280), 15.7–16.4 kDa; and H2B (P30756/B6SJZ5/C4J4M8/B4FCM8/P30755), 16.0–16.4 kDa. The labels on each 15% SDS-PAGE are as follows: MW, molecular weight markers; 1–3, sample technical replicates; H, mixture of human recombinant histones (H4, 11.4 kDa; H2B, 13.8 kDa; and H3, 15.3 kDa). ‘In solution’ indicates histones extracted into sulfuric acid followed by neutralization. ‘Precipitation’ indicates histones extracted into sulfuric acid followed by precipitation with trichloroacetic acid.

Prior to chemical derivatization, the quality of histone extracts was checked on 15% SDS-PAGE (Fig. 2). While the separation of proteins in neutralized histone extracts obtained from crude nuclei was impaired due to the natural impurities present in the plant tissues, higher-quality separation of proteins was achieved when isolated from pure, sorted nuclei, particularly with a precipitation step included. Histone proteins were highly enriched across all protocols, with the lowest proportion (∼50%) observed in Protocol 2 and the highest (∼93%) in Protocol 5 (Supplementary Fig. S1B; Supplementary Table S1).

### Selection of histone preparation protocols with the greatest potential for improved data quality

The MS data obtained from the individual protocols were compared in terms of the number of identified histone sequence variants and post-translationally modified forms of selected variants. All protocols resulted in a high number of identified sequence variants. We used the resulting data to map maize variants of histones H2A, H2B, H3, and H4, including the similarity in amino acid sequences (Supplementary Table S2). A similar portfolio of histone H2B, H3, and H4 variants was detected by all the protocols. Slight differences were observed within the H2A group, which exhibits the highest level of diversity. However, we also noted that many proteins annotated under different accession numbers corresponded to redundant or highly similar sequences and often lack experimental evidence—an issue commonly encountered in databases for non-model species such as maize. Therefore, for each protein accession number listed in Supplementary Table S2, we provide the UniProt evidence type as well as experimental support from the detected transcripts.

**Fig. 3.**
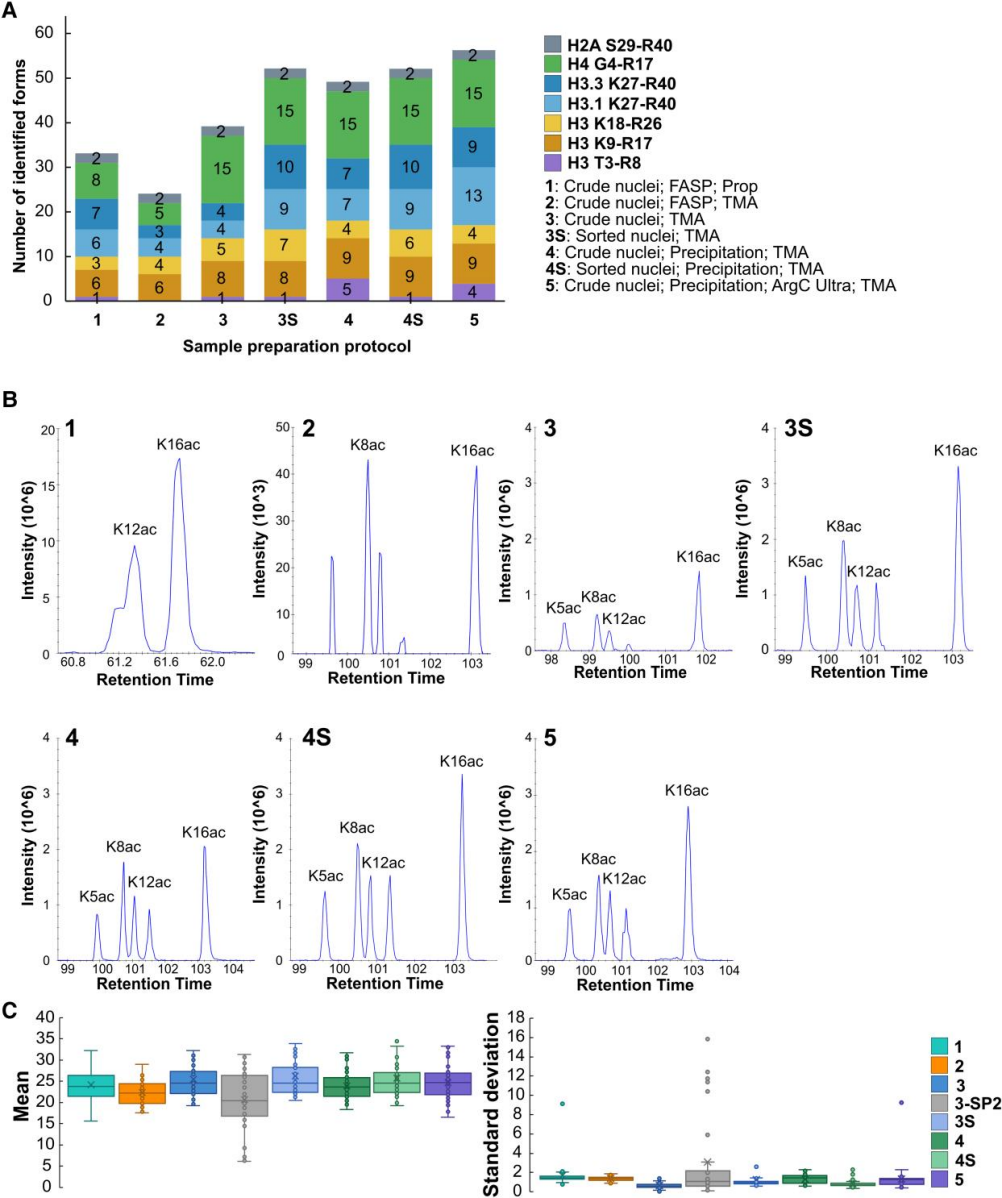
Comparison of the seven protocols tested for histone preparation. (A) The numbers of identified post-translationally modified N-terminal forms of histone H2A, H3.1, H3.3, and H4 peptides. For descriptions of the protocols see Fig. 1. (B) Representative extracted ion chromatograms (EICs) of monoacetylated N-terminal H4 G4−R17 peptidoforms for each protocol. Lysines carrying acetylation identified in particular peaks of precursor ions in the EICs are indicated. Co-elution of positional isomers occurred when propionic anhydride was used as a derivatization agent (Protocol 1; *m*/*z* 768.9465, 2+) while baseline separation was achieved using trimethylacetic anhydride (825.0091, 2+). Note that the scales for Protocols 1 and 2 differ to the other graphs. (C) The technical variability of the quantitative data in each protocol as indicated by the mean and standard deviation of the log_2_-transformed areas of the histone precursors. 3-SP2 indicates clean-up of the peptide mixture using single-pot solid-phase-enhanced peptide sample preparation on carboxylate-modified paramagnetic beads in Protocol 3.

Next, we calculated the number of identified peptidoforms of histones H2A, H3.1, H3.3, and H4 (UniProt accession nos B6T101, B6TTP0, B6UHK8, and B6T0U6, respectively; Fig. 3A). The quantitative analysis of H2B peptidoforms was not the objective of this study, since the presented protocols are not suitable for analysing chemically labelled long Arg-C-like N-terminal H2B peptides, as has previously been reported (Kuchaříková *et al*., 2021; Ryzhaya *et al*., 2025).

Altogether, 33 peptidoforms were identified using Protocol 1. The simple substitution of Prop for TMA in the FASP-based procedure (Protocol 2) reduced the number of identified forms to 24. Together with lower precursor peak intensities, this suggests that derivatization with TMA is not compatible with FASP. TMA-labelled peptides are lost during purification on the filtration unit, negating the advantages that TMA typically provides for improving peak resolution during LC and subsequent detection by MS/MS. The in-solution procedure (Protocol 3) yielded 39 histone peptidoforms but also introduced a polymeric contaminant, as observed in the total ion chromatogram of the quality control analysis (Supplementary Fig. S2). The increase in the number of peptidoforms reflected higher resolution of positional isomers in chromatographic separation, mainly those originating from histone H4 (Fig. 3B). On the other hand, fewer forms of H3.1 K27−R40 and H3.3 K27−R40 peptides were detected compared to Protocol 1. The polymeric contaminant, which generated fragment ions displaying characteristic mass differences of 44 Da, is most likely associated with the PEG backbone of residual Triton X-100, a component of the histone lysis buffer (Ahmadi and Winter, 2018). Subsequent clean-up of the peptide mixture using single-pot solid-phase-enhanced peptide sample preparation on carboxylate-modified paramagnetic beads (termed SP2) (Waas *et al*., 2019) removed the contaminant but resulted in either the loss or reduced intensities of certain precursor peaks, more than 20-fold compared to the intensities before SP2. Purified samples showed the highest variability in the standard deviation of precursor peak areas within replicates, indicating reduced reproducibility (Fig. 3C).

The sample purity was improved in Protocol 4 by adding a precipitation step prior to the derivatization, leading to an increase in the overall number of identified peptidoforms to 49. Compared with protocols 1–3, K4 occurring in a short H3 T3−R8 peptide was identified in different modified forms, including K4ac, K4me1, K4me2, and K4me3.

Replacing trypsin with newly released proteinase Arg-C Ultra allowed us to substantially simplify the protocol by omitting the protein-level derivatization step (Protocol 5). This increased the number of identified peptidoforms to 56, reflecting mainly the identification of low-abundant post-translationally modified forms of H3.1 K27−R40 and H3.3 K27−R40.

In parallel, we tested whether isolation of pure nuclei using FACS would be beneficial for histone analysis. A total of 52 post-translationally modified forms were identified using the in-solution protocol (3S) and the protocol with precipitation (4S). Surprisingly, despite otherwise valid results, we noticed the loss of modified forms of H3 T3−R8.

Except for the SP2-purified samples of Protocol 3, the variability in the standard deviation of the precursor peak areas within replicates was comparable between all the protocols tested (Fig. 3C). Protocols 2 and 3 after SP2 clean-up (3-SP2) provided the lowest median intensities of identified peptides. In particular, the median log_2_-transformed areas of precursor peaks were as follows: Protocol 1, 23.7; Protocol 2, 22.2; Protocol 3, 24.5; Protocol 3-SP2, 20.4; Protocol 4, 23.4; Protocol 5, 24.6; Protocol 3S, 24.6; and Protocol 4S, 24.6. Based on the results obtained, Protocols 4, 5, 3S, and 4S with the highest number of identified peptidoforms, the highest quality of chromatographic separation, and the highest precursor peak areas were selected for the detailed inspection of the quantitative data.

### Performance of Protocols 4, 5, 3S, and 4S in quantitative terms

The relative abundances of histone N-terminal peptidoforms were used to compare the quantitative data between the protocols. The dataset of each protocol was evaluated independently, with the internal identification alignment across the raw files based on retention time and *m*/*z*. The quantities of histone H2A, H3, and H4 peptidoforms were determined from peak areas in EICs. The relative abundances of individual histone peptidoforms were calculated as the ratio of their EIC precursor peak area to the sum of the EIC areas of all detected forms of the respective peptide sequence.

**Fig. 4.**
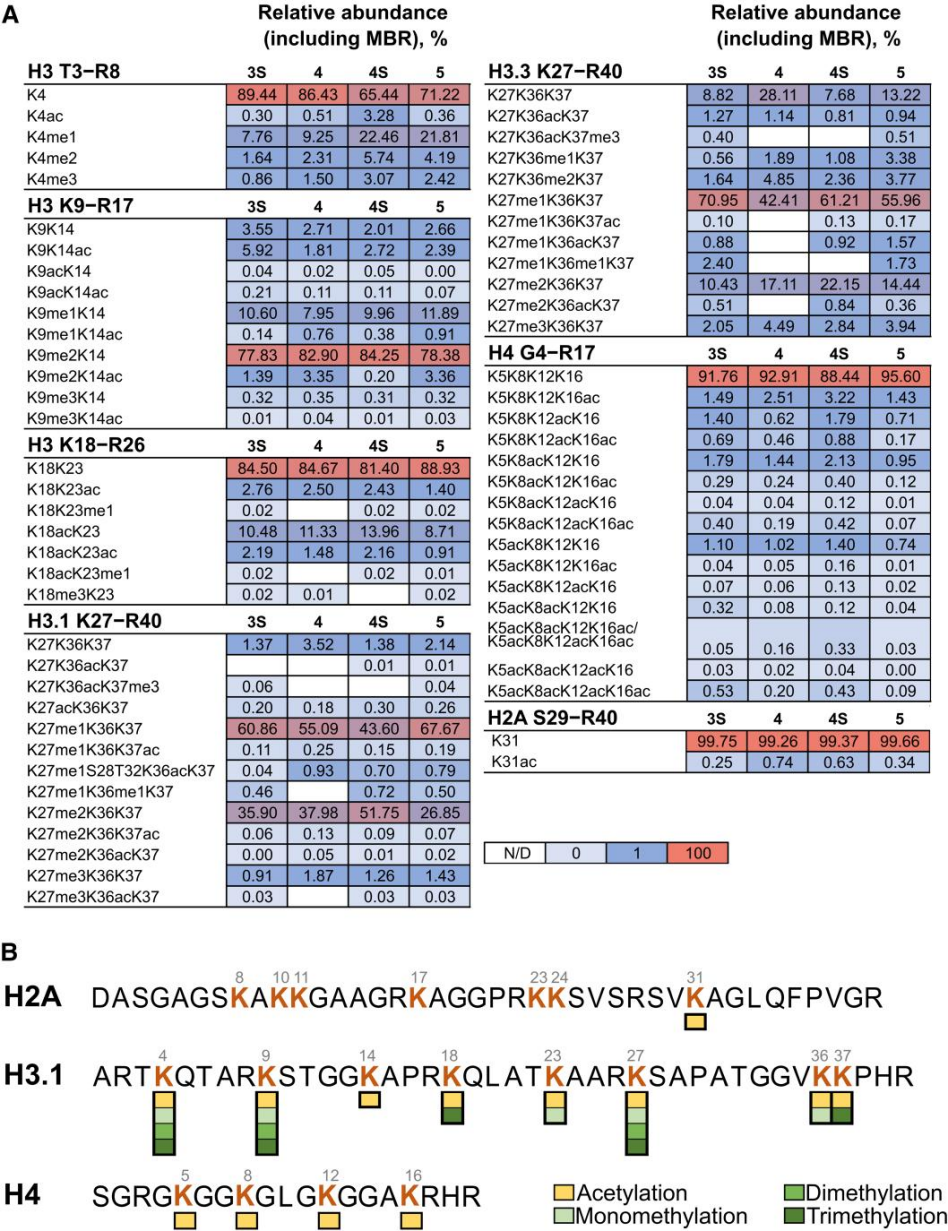
Post-translational modifications (PTMs) quantified in histone H2A, H3, and H4 peptides prepared using Protocols 3S, 4, 4S, and 5 (Fig. 1). (A) Quantitative results with ‘match-between-runs’ (MBR) applied. The relative abundances of individual histone peptidoforms were calculated as the ratio of their EIC precursor peak area to the sum of the EIC areas of all detected forms of the respective peptide sequence. N/D, not detected. For a comparison of quantitative data with and without MBR, see Supplementary Fig. S3. Note that data without MBR are used for Fig. 3. (B) Overview of PTMs identified in H2A, H3, and H4 N-termini using Protocols 3S, 4, 4S, and 5. Comparison of the combinatorial pattern of PTMs detected in Protocols 1, 3S, and 5 is depicted in Supplementary Fig. S4.

The relative quantitative data related to histone H4 were consistent across the protocols (Fig. 4A). The results of all the protocols confirmed that the non-modified form of the G4‒R17 peptide was highly dominant with a relative abundance of around 90%. The relative abundance of mono-acetylated forms corresponded to 1–3%, while the abundance of all multiple acetylated forms was less than 1%. In histone H3 peptides, the dominating forms were non-modified T3‒R8 and K18‒R26, K9‒R17 with K9me2, and K27‒R40 with K27me1. Histone H2A is generally less extensively post-translationally modified compared with H3 and H4. In the S29‒R40 peptide analysed here, the non-modified form was predominant, with the acetylated form detected at levels below 1%.

The higher number of missing values in the histone H3 data across protocols prompted us to investigate whether these were caused by missed fragmentation or an absence of detectable precursors. Because DDA targets only the most intense precursors in each cycle, precursor selection can vary between runs due to stochastic fluctuations in ion intensity and minor changes in ionization efficiency. As noted above, the absence of post-translationally modified H3 T3‒R8 forms was most striking. The complete set of peptides, that is unmodified K4, K4ac, K4me1, K4me2, and K4me3, was identified only in Protocol 4. Four peptidoforms were identified in Protocol 5, with dimethylated lysine missing. Only the unmodified form was detected in the samples prepared from the sorted nuclei (Protocols 3S and 4S). On the other hand, certain peptidoforms identified in Protocols 3S and 4S (e.g. H3K18‒R26 with K23me1 or H3.3K27‒R40 with K27me3) were missing in Protocols 4 or 5 (Supplementary Fig. S3). A deeper inspection of the data revealed that the absence of modified forms was often caused by missed fragmentation. To mitigate missing data resulting from this fragmentation issue, predefined Skyline spectral libraries with expected modified sequences are routinely employed. Therefore, we searched for missing peaks with the corresponding *m*/*z* and retention times using spectral libraries from other protocols in which they were identified (hereafter referred to as ‘match-between-runs’, MBR) and quantified them to compare the approaches using complete datasets (Fig. 4; Supplementary Fig. S3). PTMs identified in the N-terminal regions of maize histones H3, H4, and H2A using protocols 3S, 4, 4S, and 5 are summarized in Fig. 4B.

## Discussion

The study of the chromatin landscape has become a popular area of biological research due to its direct link to gene regulation. Although chromatin remodelling mechanisms are widely conserved among eukaryotes, the capacity of plants to adapt to continuously varying environmental conditions suggests higher epigenetic flexibility than in animals (Probst *et al*., 2020). Alongside RNA molecules and DNA methylation, histone sequence variants with their complex and combinatorial character of PTMs provide an efficient regulatory mechanism for maintaining chromosomal structure within cells (reviewed by Candela-Ferre *et al*., 2024).

Mass spectrometry is particularly helpful in investigating the roles of histones in chromatin dynamics. Compared with traditional approaches, it allows for the extraction of comprehensive information on the types of histone marks and their positions in amino acid sequences from a single analysis. Importantly, it also enables the detection of combinatorial patterns of PTMs on individual peptides. However, beyond the common challenges of MS-based histone analysis (Maile *et al*., 2015; Sidoli *et al*., 2015; Kuchaříková *et al*., 2021), research on plant histones is further complicated by the species-specific chemical composition of plant tissues. This limitation is reflected in the relatively small number of studies addressing plant histone epigenetics (Johnson *et al*., 2004; Chen *et al*., 2015; Mahrez *et al*., 2016; Jamge *et al*., 2023). It has been shown that the clean-up of plant histone extracts on cut-off membranes coupled with Prop derivatization can serve as a relatively robust and versatile method for a variety of plant tissues, including seedlings, leaves, siliques, and calli of Arabidopsis, leaves of *Solanum lycopersicum*, *Nicotiana benthamiana*, and *Zea mays* (Kuchaříková *et al*., 2022). However, the use of Prop represents a limitation in terms of insufficient hydrophobicity for chromatographic peak resolution of many histone peptidoforms. The aim of this study was therefore to broaden the applicability of bottom-up histone proteomics in plant research.

Notably, the quality and reliability of MS data depend on the multi-step workflow involved in histone characterization. In this study, we focused on optimizing histone purity, enzymatic digestion, and chemical derivatization of amine groups to enhance the quality of data obtained by subsequent LC-MS/MS analysis. We introduced TMA-labelling to plant histone preparation workflows before LC-MS/MS (Fig. 1), with the aim of increasing the number of identified and quantified post-translationally modified histone forms by enhancing peptidoform hydrophobicity and the resolution of positional isomers. Replacing Prop with TMA in filter-aided histone preparation yielded a lower number of identified peptidoforms (Fig. 3A). Conversely, direct derivatization of the histone extract using TMA confirmed the necessity of clean-up of the crude plant histones before LC-MS/MS analysis. The strong compositional bias in amino acid content (i.e. the over-representation of basic and the under-representation of acidic, aromatic, and cysteine residues; Müller and Muir, 2015) predetermines the specific behaviour of histone proteins and peptides towards the solvents and sorbents used for sample clean-up (Fig. 3). Purification at the protein level appears to be a more reliable approach, as purifying chemically derivatized histone peptides resulted in substantial selective losses of post-translationally modified forms. The selection of purification methods for proteins and peptides always requires careful consideration, as every extra step potentially decreases the number of identified peptides and/or their quantity. This is especially problematic for the characterization of histones, where a highly covered sequence is a prerequisite for the identification of histone marks in specific amino acid sites and histone sequence variants based on their unique peptides.

As reported previously, purifying histones from Arabidopsis leaves by precipitation leads to problematic re-solubilization, and is not efficient enough as the samples require further methanol:chloroform clean-up (Scheid *et al*., 2022). Species-specific levels of polyphenols, polysaccharides, lipids, and oxidative enzymes can co-precipitate with proteins or promote cross-linking, forming densely aggregated or chemically modified precipitates that are inherently difficult to solubilize. Consequently, protein re-solubilization efficiency varies among plant species. Here, we show that histone re-solubilization is not limiting in maize, indicating that precipitation alone can provide an adequate purification step prior to LC-MS/MS. Indeed, precipitation followed by Arg-C Ultra digestion and peptide-level TMA labelling (Protocol 5) resulted in the detection of a high number of histone peptidoforms (Fig. 3A), demonstrating that Arg-C Ultra cleavage is advantageous for plant histones. This proves that the preparation of plant histones can be simplified by omitting derivatization at the protein level, analogous to the workflows used for mammalian samples (Ryzhaya *et al*., 2025).

In-solution derivatization can be performed directly on histones extracted from pure nuclei isolated by FACS, providing a strategy to eliminate tissue-derived compounds interfering with MS and yielding higher protein amounts compared with extracts from crude nuclei preparations. This approach offers a viable alternative for histone preparation in cases where the chemical composition of plant tissues affects histone re-solubilization or interferes with chemical derivatization. Overall, FACS-based nuclei purification is a versatile method applicable to all plant species whose cells are prone to lysis and that can provide a sufficient amount of tissue for analysis. Obviously, sorting nuclei from small plants or specific organs might be inefficient due to the large number of nuclei required and the associated laboratory and time demands. Consequently, proteomic analysis of the model plant Arabidopsis employs protocols that do not involve flow cytometry.

Limited information on histone sequence variants and their modification status is available for crop plants (Casati, 2012). To our knowledge, we present a first MS-based approach for profiling histone sequence variants and their PTMs in maize. Compared to Arabidopsis (Franek *et al*., 2024), less acetylated histone H4 and a higher percentage of H3K9me2 were detected (Fig. 4), suggesting a more compact chromatin structure in maize. Together with a higher number of histone variants, whose biological roles remain largely unexplored even in Arabidopsis, this highlights the vast potential for uncovering mechanisms of chromatin dynamics in crop plants. The study of plant histone epigenetics is currently limited by the availability of methodology, by the species-specific composition of plant cells, and by the amount of plant material available for histone extraction. An improved methodology is required to enable the study of histones across diverse crop species and to enable the transfer of findings on histone PTMs to plant breeding programs. From this perspective, TMA-based derivatization methods have the potential to advance our understanding of chromatin regulatory mechanisms in crop plants. Based on the protocols tested, we recommend either precipitation followed by Arg-C Ultra digestion (Protocol 5) or nuclear sorting followed by in-solution derivatization (Protocol 3S) as the preferred methods for histone analysis in plants. These approaches yielded a greater number of peptides with diverse combinatorial PTM patterns compared to the FASP-based procedure (Supplementary Fig. S4). The effectiveness of Arg-C Ultra proteinase supports omitting the protein-level derivatization, and we therefore propose its use as a general replacement of trypsin across protocols. A combined workflow using Arg-C Ultra digestion and TMA-based chemical derivatization enables broad characterization of H2A, H3, and H4 histone sequence variants, with TMA improving chromatographic retention and separation of short peptides. In contrast, derivatization should be omitted for longer Arg-C-like peptides containing multiple lysines (such as the N-terminal peptides of H2B variants, which have at least 10 lysine residues) because TMA-labelling causes excessively strong retention, preventing some peptides from eluting within the applied gradient (Supplementary Fig. S5).

Overall, the protocols presented here can enable robust comparison of the relative abundances of multiple histone PTMs across different growth conditions, genetic backgrounds, and cell types. Beyond standard histone extraction, the workflow is also compatible with pull-down assays, allowing the characterization of PTMs in tagged histone variants. Although tested on *Zea mays*, the protocols can be readily adapted for a wide range of crop species. When transferring the workflow to other plants, particular attention should be paid to achieving efficient cell disruption, ensuring nuclei/histone extract purity and complete re-solubilization of the histone extract if precipitation is included, and performing careful data post-processing. Finally, histone characterization in crops remains challenged by sequence redundancy in available databases, highlighting the need for improved genome annotations to support future PTM studies.

## Supplementary Material

erag100_Supplementary_Data

## Data Availability

Protocol 3S and Protocol 5 in step-by-step form are available at Zenodo https://doi.org/10.5281/zenodo.15877728; Ryzhaya *et al*. (2026a). The MS data and the three databases used for searches (in-house histone *Zea mays* database v230721; UniProt KB *Zea mays* database v231108; and the modified cRAP contamination database) are also available at Zenodo https://doi.org/10.5281/zenodo.15877450; Ryzhaya *et al*. (2026b).

## Supplementary data

The following supplementary data are available at *JXB* online.

Fig. S1. Quality control of samples prepared using the different protocols.

Fig. S2. Total ion chromatograms showing a contaminant in the sample prepared using Protocol 3.

Fig. S3. Quantitative results of histone H2A, H3, and H4 peptides prepared using Protocols 3S, 4, 4S, and 5 with and without MBR applied.

Fig. S4. Diagram illustrating the advantages of Protocols 3S and 5 over Protocol 1, which is currently used for plant histone preparation.

Fig. S5. Diagram illustrating challenges in the characterization of histone H2B.

Table S1. Peptidoforms, source proteins, and protein groups detected using each protocol.

Table S2. Histone sequence variants identified in *Zea mays* using the different protocols.
