# Supplementary material for: Enhanced plant bottom-up histone proteomics
Source: J Exp Bot. 2026 Feb 24;77(12):3581–92. doi: 10.1093/jxb/erag100 (PMC13293081; doi:10.1093/jxb/erag100)
Supplement: erag100_Supplementary_Data [file erag100_supplementary_data.zip › jexbot316775-file001.pdf]

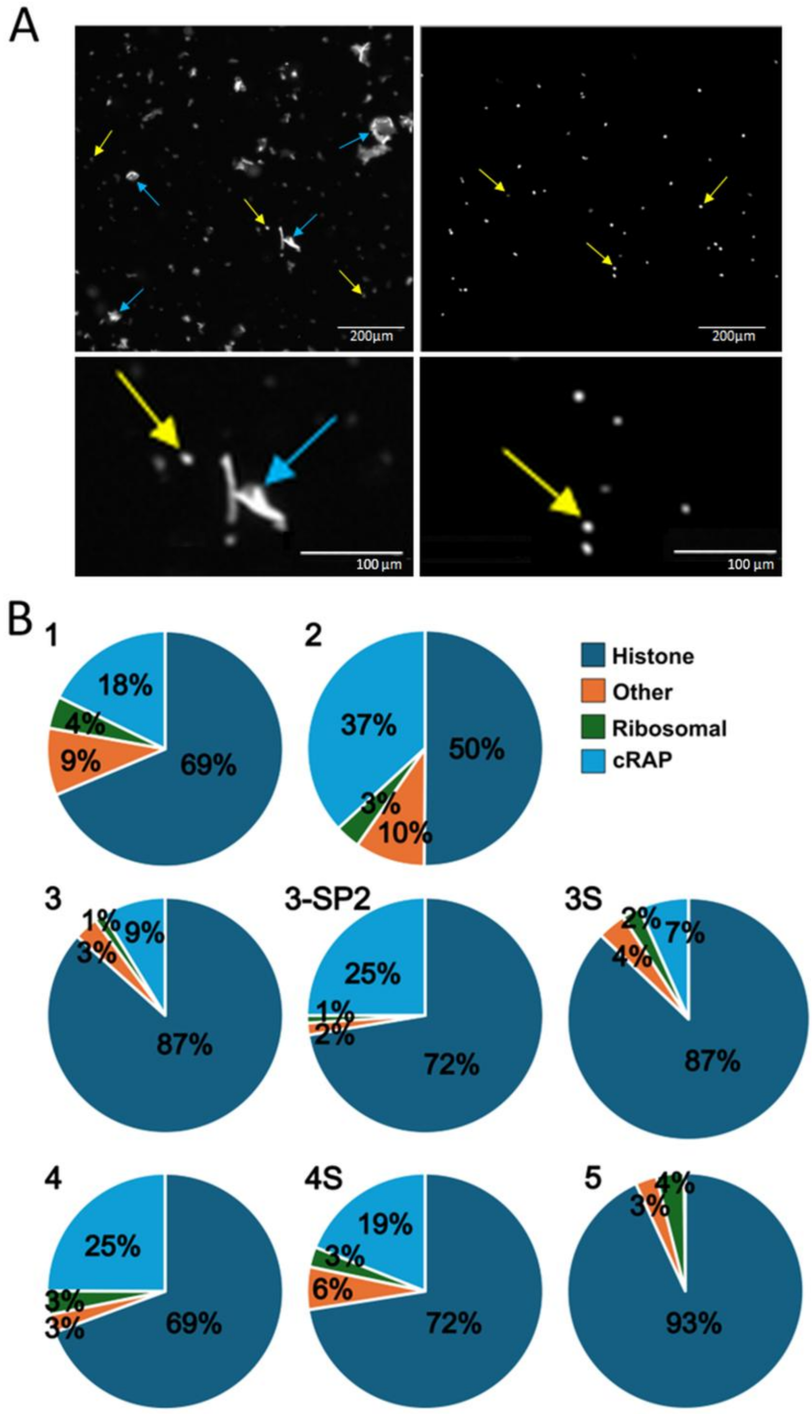

Figure S1. Quality control of samples prepared by different protocols. A) Nuclei sorted by FACS (right) are highly pure, in contrast to crude nuclei isolated by Percoll gradient centrifugation (left), which remain contaminated with tissue debris. Representative nuclei and debris particles are indicated by yellow and blue arrows, respectively. The lower panel shows a 300% magnified view. B) Proportions of histones vs. contaminating non-histone proteins based on precursor peak intensities. Three main factors appear to influence the purity and quantity of identified histone peptides: the number of sample preparation steps, peptide adsorption to surfaces, and the impact of sample characteristics on ionization efficiency. The highest amounts of histone peptides were obtained using protocols 3, 3S and 5. The highest proportion of non-histone and cRAP–derived proteins were detected in protocol 2, due to the TMA-labelled histone losses on the filter unit. Ribosomal proteins are shown as a separate group, as they are common non-histone contaminants in histone extracts.

Before SP2

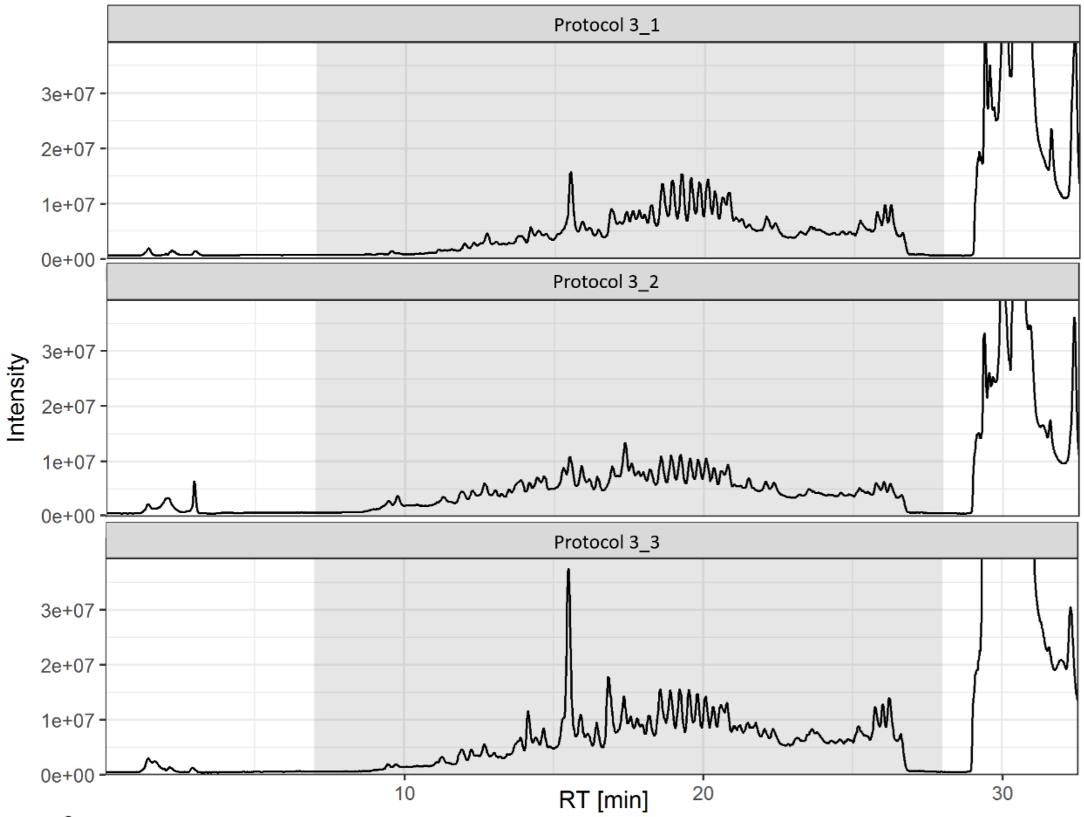

After SP2

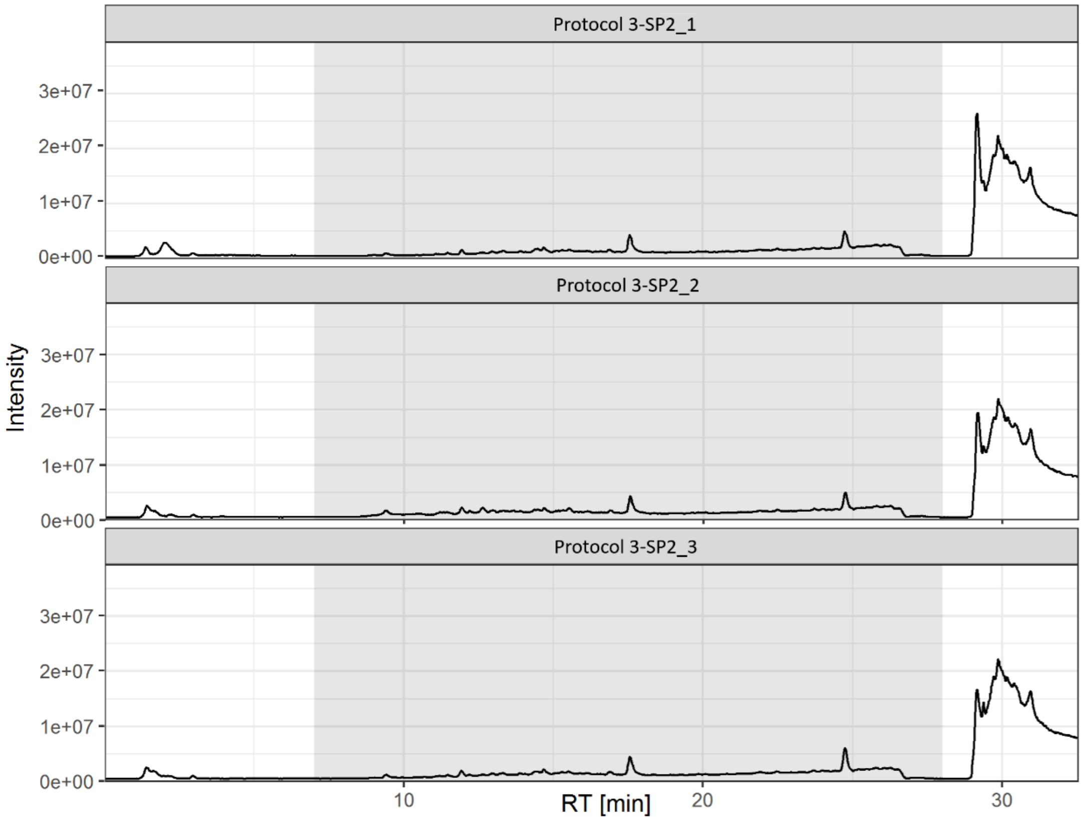

Figure S2. Total Ion Chromatogram showing a contaminant in the sample prepared by protocol 3 (i.e., crude nuclei; TMA). The chromatogram below shows contaminant removal using SP2 protocol. A ten-times diluted sample was used for quality control, and the measurement was performed using an MS Impact II (Bruker).

	Relative abundance, %				Relative abundance (including MBR), %			
**H3 T3−R8**	**3S**	**4**	**4S**	**5**	**3S**	**4**	**4S**	**5**
K4	100.00	86.43	100.00	74.34	89.44	86.43	65.44	71.22
K4ac		0.51		0.37	0.30	0.51	3.28	0.36
K4me1		9.25		22.76	7.76	9.25	22.46	21.81
K4me2		2.31			1.64	2.31	5.74	4.19
K4me3		1.50		2.53	0.86	1.50	3.07	2.42
**H3 K9−R17**	**3S**	**4**	**4S**	**5**	**3S**	**4**	**4S**	**5**
K9K14	3.55	2.71	2.01	2.66	3.55	2.71	2.01	2.66
K9K14ac	5.92	1.81	2.72	2.39	5.92	1.81	2.72	2.39
K9acK14	0.04		0.05	0.00	0.04	0.02	0.05	0.00
K9acK14ac	0.21	0.11	0.11	0.07	0.21	0.11	0.11	0.07
K9me1K14	10.60	7.95	9.96	11.89	10.60	7.95	9.96	11.89
K9me1K14ac	0.14	0.76	0.38	0.91	0.14	0.76	0.38	0.91
K9me2K14	77.83	82.92	84.26	78.40	77.83	82.90	84.25	78.38
K9me2K14ac	1.39	3.35	0.20	3.36	1.39	3.35	0.20	3.36
K9me3K14	0.32	0.35	0.31	0.32	0.32	0.35	0.31	0.32
K9me3K14ac	0.01	0.04			0.01	0.04	0.01	0.03
**H3 K18−R26**	**3S**	**4**	**4S**	**5**	**3S**	**4**	**4S**	**5**
K18K23	84.50	84.68	81.40	88.89	84.50	84.67	81.40	88.93
K18K23ac	2.76	2.50	2.43	1.41	2.76	2.50	2.43	1.40
K18K23me1	0.02		0.02		0.02		0.02	0.02
K18acK23	10.48	11.33	13.96	8.78	10.48	11.33	13.96	8.71
K18acK23ac	2.19	1.48	2.16	0.92	2.19	1.48	2.16	0.91
K18acK23me1	0.02		0.02		0.02		0.02	0.01
K18me3K23	0.02				0.02	0.01		0.02
**H3.1 K27−R40**	**3S**	**4**	**4S**	**5**	**3S**	**4**	**4S**	**5**
K27K36K37	1.38	3.56	1.39	2.14	1.37	3.52	1.38	2.14
K27K36acK37				0.01			0.01	0.01
K27K36acK37me3				0.04	0.06			0.04
K27acK36K37	0.20	0.18	0.31	0.26	0.20	0.18	0.30	0.26
K27me1K36K37	61.18	55.68	43.93	67.67	60.86	55.09	43.60	67.67
K27me1K36K37ac	0.11	0.25	0.15	0.19	0.11	0.25	0.15	0.19
K27me1S28T32K36acK37	0.04		0.71	0.79	0.04	0.93	0.70	0.79
K27me1K36me1K37				0.50	0.46		0.72	0.50
K27me2K36K37	36.09	38.28	52.14	26.85	35.90	37.98	51.75	26.85
K27me2K36K37ac	0.06	0.16	0.09	0.07	0.06	0.13	0.09	0.07
K27me2K36acK37				0.02	0.00	0.05	0.01	0.02
K27me3K36K37	0.91	1.89	1.27	1.43	0.91	1.87	1.26	1.43
K27me3K36acK37	0.03		0.03	0.03	0.03		0.03	0.03
**H3.3 K27−R40**	**3S**	**4**	**4S**	**5**	**3S**	**4**	**4S**	**5**
K27K36K37	8.83	28.11	7.68	14.26	8.82	28.11	7.68	13.22
K27K36acK37	0.40	1.14	0.81	1.02	1.27	1.14	0.81	0.94
K27K36acK37me3	0.56			0.55	0.40			0.51
K27K36me1K37	1.27	1.89	1.08	3.64	0.56	1.89	1.08	3.38
K27K36me2K37	1.64	4.85	2.36	4.07	1.64	4.85	2.36	3.77
K27me1K36K37	71.01	42.41	61.21	60.33	70.95	42.41	61.21	55.96
K27me1K36K37ac			0.13	0.19	0.10		0.13	0.17
K27me1K36acK37	0.88		0.92		0.88		0.92	1.57
K27me1K36me1K37	2.40				2.40			1.73
K27me2K36K37	10.44	17.11	22.15	15.57	10.43	17.11	22.15	14.44
K27me2K36acK37	0.51		0.84	0.39	0.51		0.84	0.36
K27me3K36K37	2.06	4.49	2.84		2.05	4.49	2.84	3.94
**H4 G4−R17**	**3S**	**4**	**4S**	**5**	**3S**	**4**	**4S**	**5**
K5K8K12K16	91.76	92.91	88.44	95.56	91.76	92.91	88.44	95.60
K5K8K12K16ac	1.49	2.51	3.22	1.43	1.49	2.51	3.22	1.43
K5K8K12acK16	1.40	0.62	1.79	0.71	1.40	0.62	1.79	0.71
K5K8K12acK16ac	0.69	0.46	0.88	0.17	0.69	0.46	0.88	0.17
K5K8acK12K16	1.79	1.44	2.13	0.95	1.79	1.44	2.13	0.95
K5K8acK12K16ac	0.29	0.24	0.40	0.12	0.29	0.24	0.40	0.12
K5K8acK12acK16	0.04	0.04	0.12	0.01	0.04	0.04	0.12	0.01
K5K8acK12acK16ac	0.40	0.19	0.42	0.07	0.40	0.19	0.42	0.07
K5acK8K12K16	1.10	1.02	1.40	0.74	1.10	1.02	1.40	0.74
K5acK8K12K16ac	0.04	0.05	0.16	0.01	0.04	0.05	0.16	0.01
K5acK8K12acK16	0.07	0.06	0.13	0.02	0.07	0.06	0.13	0.02
K5acK8acK12K16	0.32	0.08	0.12	0.04	0.32	0.08	0.12	0.04
K5acK8acK12K16ac/ K5acK8K12acK16ac	0.05	0.16	0.33	0.06	0.05	0.16	0.33	0.03
K5acK8acK12acK16	0.03	0.02	0.04	0.00	0.03	0.02	0.04	0.00
K5acK8acK12acK16ac	0.53	0.20	0.43	0.09	0.53	0.20	0.43	0.09
**H2A S29−R40**	**3S**	**4**	**4S**	**5**	**3S**	**4**	**4S**	**5**
K31	99.74	99.15	99.37	99.66	99.75	99.26	99.37	99.66
K31ac	0.26	0.85	0.63	0.34	0.25	0.74	0.63	0.34

N/D	0	1	100

Figure S3. Quantitative results of histone H2A, H3 and H4 peptides prepared by protocols 3S, 4, 4S, and 5 without (left) and with MBR (right) applied. Relative abundance corresponds to the percentage of individual peptide form peak area of EIC from the sum of the EIC areas of all forms of the respective peptide sequence. N/D – Not Detected.

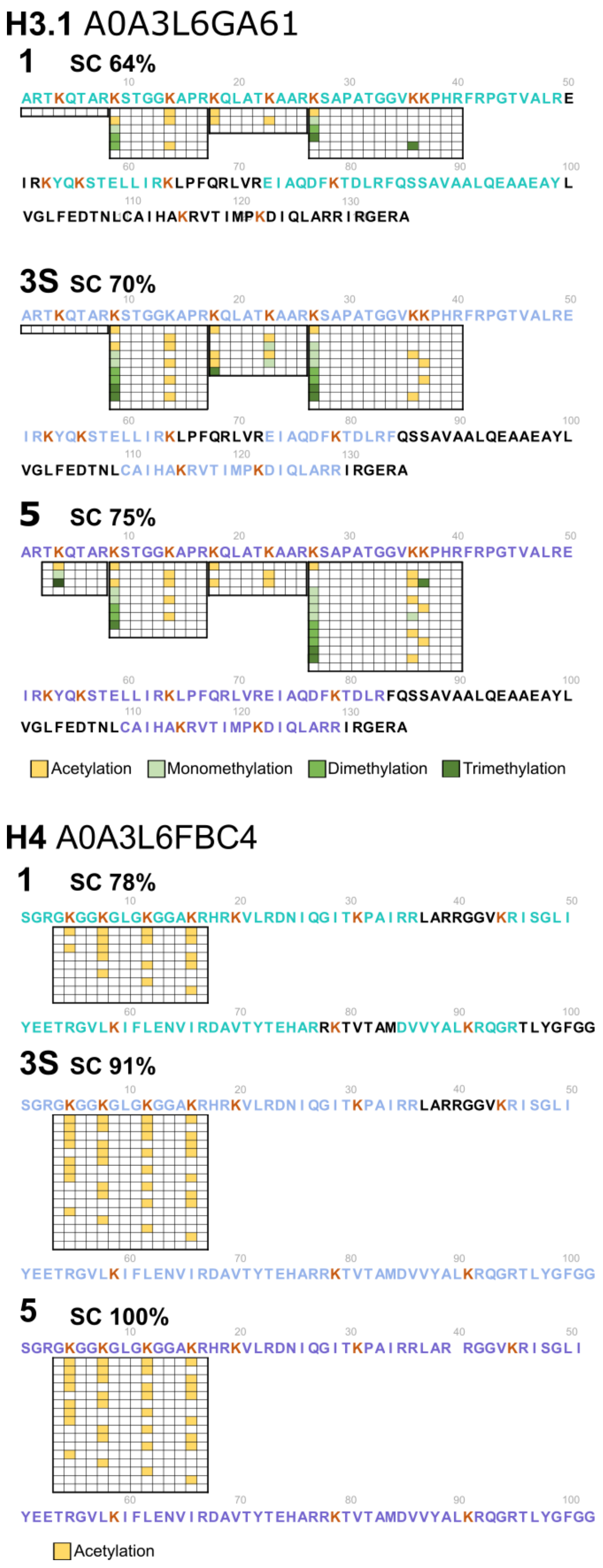

Figure S4. The scheme illustrating the advantages of protocols 3S and 5 over protocol 1, which is currently used for plant histone preparation. Sequence coverage (SC) is indicated by colored letters, while black letters represent parts of sequences that have not been identified. The grids below the respective sequences depict the combinatorial patterns identified for peptides of the H3.1 and H4 N-termini.

H2B B6U2K4

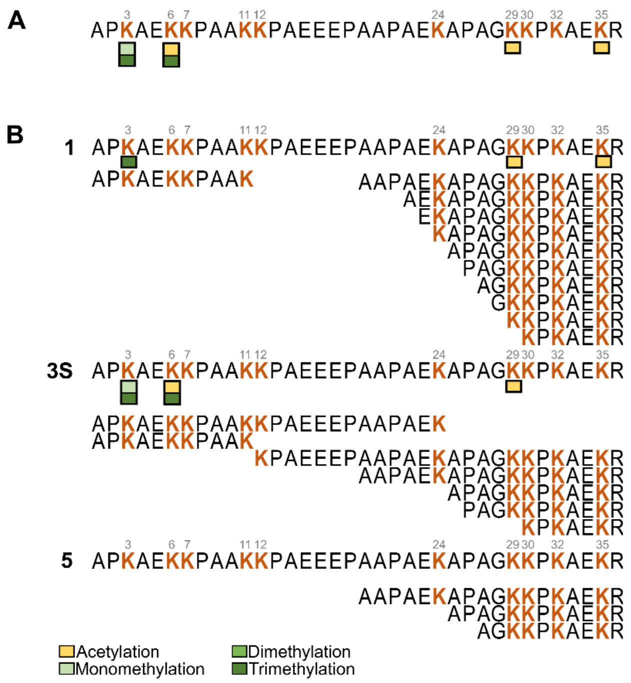

Figure S5. Scheme illustrating challenges in the characterization of histone H2B. (A) Overview of PTMs identified in the H2B N-terminal region. (B) Comparison of PTM patterns obtained using protocols 1, 3S, and 5. Due to the presence of ten TMA-labeled lysines, the highly hydrophobic desired N-terminal sequence (A1–R36) was not eluted within the applied LC gradient. Consequently, the PTMs shown were observed in shorter peptides resulting from incorrect protein digestion (as depicted below the desired sequence). For this reason, analysis of H2B proteins digested with Arg-C Ultra, without chemical derivatization, is more suitable.
